# Traditional healers, faith healers and medical practitioners: the contribution of medical pluralism to bottlenecks along the cascade of care for HIV/AIDS in Eastern and Southern Africa

**DOI:** 10.1136/sextrans-2016-052974

**Published:** 2017-07-23

**Authors:** Mosa Moshabela, Dominic Bukenya, Gabriel Darong, Joyce Wamoyi, Estelle McLean, Morten Skovdal, William Ddaaki, Kenneth Ondeng’e, Oliver Bonnington, Janet Seeley, Victoria Hosegood, Alison Wringe

**Affiliations:** 1 Department of Rural Health, School of Nursing and Public Health, University of KwaZulu-Natal, Durban, South Africa; 2 Africa Health Research Institute, KwaZulu-Natal, South Africa; 3 MRC/UVRI Uganda Research Unit on AIDS, Entebbe, Uganda; 4 Tanzanian National Institute of Medical Research, Mwanza, Tanzania; 5 Malawi Epidemiology and Intervention Research Unit, Karonga, Malawi; 6 London School of Hygiene and Tropical Medicine, London, UK; 7 Department of Public Health, University of Copenhagen, Copenhagen, Denmark; 8 Biomedical Research and Training Institute, Harare, Zimbabwe; 9 Rakai Health Sciences Program, Rakai, Uganda; 10 Kenya Medical Research Institute, Centre for Global Health Research, Kisumu, Kenya; 11 University of Southampton, Southampton, UK

**Keywords:** Medical Pluralism, HIV, Health-seeking behaviour, Traditional healer, Faith healer, Antiretroviral treatment, Sub-Saharan Africa

## Abstract

**Objectives:**

There are concerns that medical pluralism may delay patients’ progression through the HIV cascade-of-care. However, the pathways of impact through which medical pluralism influence the care of people living with HIV (PLHIV) in African settings remain unclear. We sought to establish the manifestation of medical pluralism among PLHIV, and explore mechanisms through which medical pluralism contributes bottlenecks along the HIV care cascade.

**Methods:**

We conducted a multicountry exploratory qualitative study in seven health and demographic surveillance sites in six eastern and southern African countries: Uganda, Kenya, Tanzania, Malawi, Zimbabwe and South Africa. We interviewed 258 PLHIV at different stages of the HIV cascade-of-care, 48 family members of deceased PLHIV and 53 HIV healthcare workers. Interviews were conducted using shared standardised topic guides, and data managed through NVIVO 8/10/11. We conducted a thematic analysis of healthcare pathways and bottlenecks related to medical pluralism.

**Results:**

Medical pluralism, manifesting across traditional, faith-based and biomedical health-worlds, contributed to the care cascade bottlenecks for PLHIV through three pathways of impact. First, access to HIV treatment was delayed through the nature of health-related beliefs, knowledge and patient journeys. Second, HIV treatment was interrupted by availability of alternative options, perceived failed treatment and exploitation of PLHIV by opportunistic traders and healers. Lastly, the mixing of biomedical healthcare providers and treatment with traditional and faith-based options fuelled tensions driven by fear of drug-to-drug interactions and mistrust between providers operating in different health-worlds.

**Conclusion:**

Medical pluralism contributes to delays and interruptions of care along the HIV cascade, and mistrust between health providers. Region-wide interventions and policies are urgently needed in sub-Saharan Africa to minimise potential harm and consequences of medical pluralism for PLHIV. The role of sociocultural beliefs in mediating bottlenecks necessitate adoption of culture-sensitive approaches intervention designs and policy reforms appropriate to the context of sub-Saharan Africa.

## Introduction

For more than 30 years, there have been calls for collaboration between the traditional and biomedical health systems in order to address the health problems and disparities of people in sub-Saharan Africa.^1^ A collection of best practice examples on ways in which traditional healers could collaborate with biomedical providers in the prevention of HIV infection and provision of care for people living with HIV (PLHIV) in sub-Saharan Africa was published by the United Nations Programme on HIV/AIDS a decade ago.^2^ Yet, no country in sub-Saharan Africa has an integrated or collaborative healthcare system that recognises both traditional and biomedical approaches.^3^ These plural healthcare systems continue to function in a fragmented manner, thereby exacerbating access barriers to healthcare in sub-Saharan Africa, arguably best observed through practices of medical pluralism among PLHIV.^3 4^

Medical pluralism is defined as the use of more than one medical system for health and illness.^5^ In sub-Saharan Africa, medical pluralism involves ‘shopping and switching’ between multiple modalities of care available,^6 7^ including traditional and faith-based healthcare systems.^4^ The utilisation of fragmented, plural medical systems has been repeatedly shown to delay access to HIV testing and antiretroviral treatment (ART) initiation among PLHIV when their pathways of care include traditional and faith healers.^6 8 9^ These delays may occur because some PLHIV seek comfort and sanctuary among traditional and faith healers.^10^ As high as one in five ART users consult traditional healers,^8^ with the use of traditional healers at times associated with unsuppressed viral loads and treatment interruptions.^11^ PLHIV incur excessive out-of-pocket costs, often at catastrophic household expenditure levels, in order to fulfil their healthcare needs through medical pluralism.^12 13^ Despite these negative effects, medical pluralism remains a highly prevalent form of healthcare behaviour in sub-Saharan Africa.^14 15^

The last decade has seen a growing body of literature on the study of medical pluralism, related in particular to the need to strengthen weak health systems and curb the epidemic of HIV/AIDS in sub-Saharan Africa.^4^ However, there are currently no studies that have examined the role of medical pluralism along the biomedically inclined ‘cascade-of-care’ for PLHIV. Furthermore, medical pluralism studies tend to be localised, and for this reason, medical pluralism is yet to be recognised as a generalised research problem of regional importance throughout sub-Saharan Africa. This study explores the contribution of medical pluralism to bottlenecks occurring along the cascade-of-care for PLHIV in six sub-Saharan Africa countries. Specifically, the study aims to explore the manifestation of medical pluralism throughout the HIV care continuum, and mechanisms through which medical pluralism influence bottlenecks to care and treatment across multiple countries and healthcare systems in eastern and southern Africa.

## Methods

### Study design and setting

A multi-country exploratory qualitative study was conducted in seven health and demographic surveillance studies (HDSS) located in six countries, all participating members of the Analysing Longitudinal Population-based HIV/AIDS data on Africa network; Kisumu, Kenya; Kyamulibwa and Rakai, Uganda; Kisesa, Tanzania; Karonga, Malawi; Manicaland, Zimbabwe and uMkhanyakude, South Africa.^16^ Details about the methods used to conduct the study in each site have been described in full in the supplementary methods of the editorial at http://dx.doi.org/10.1136/sextrans-2017-053172.

### Study sampling and participants

We recruited PLHIV, sampled along the stages of the cascade-of-care for HIV, including those who were diagnosed with HIV but not engaged in care, pre-ART engaged in care, those in different stages of ART care and those who dropped out of ART care. Healthcare workers (HCWs) involved in HIV care were recruited, as were family members of people who died with a known diagnosis of HIV. A combination of methods was used to recruit participants, including through databases kept by HDSS and registers in HIV and ART clinics, and potential participants were approached through home visits, in clinics and by telephone. The verbal autopsy platform in HDSS, normally used to interview family members of people who have died for the purposes of establishing possible causes of death, was used to identify deceased HIV patients and their family members. While purposive sampling was the main sampling technique used, random and snowballing techniques were also used, explained further elsewhere.^17^ The study included a total of 53 HCWs, 48 family members of the deceased with a known diagnosis HIV and 258 PLHIV. PLHIV were sampled by pre-ART (81), ART (137) and lost-to-follow-up (40) categories across seven HDSS.

### Data collection and management

In-depth interviews with study participants were conducted by trained and experienced qualitative interviewers, using shared topic guides across study settings. Data collection tools and topics were tailored to the type of participant being interviewed, such as PLHIV or HCW, but common across all study settings. Interviews were conducted in the preferred language of the participant, and a private venue of their choice, often their own homes or rooms in clinics. Patient journeys through healthcare were retrospectively charted during data collection, documenting key events and their respective dates. All interviews were audio-recorded, and field notes were taken. Audio-recordings were also transcribed verbatim and translated into English, and transcripts were managed through NVIVO 8/10/11 software, but manually in Kyamulibwa.^17^

### Data analysis and interpretation

Data were analysed thematically to generate themes and subthemes. Participant transcripts were coded individually in respective countries using inductive open coding, and coded data sent to a central location for further analysis. Pathways through healthcare were coded first to identify sources of healthcare, followed by themes related to medical pluralism as a bottleneck in the HIV care cascade of PLHIV who engaged in medical pluralism. Axial coding techniques were used for thematic analysis of data repetitiveness, similarities and differences, comparing coded transcripts within and across countries and within and across stages of the cascade. Codes were analysed for relatedness, in order to generate categories. Codes were revisited to identify contradictory and deviant codes, followed by revision of codes, categories and themes. Themes and subthemes are presented in the results section, but participant identifiers have been removed for anonymity.

### Ethical considerations

Ethical approval was obtained through certified research committees in all participating countries, and from the London School of Hygiene and Tropical Medicine (#10389). Informed consent was requested and obtained from all participants.

## Results

Three thematic areas emerged, through which medical pluralism contributed to bottlenecks along the cascade-of-care for PLHIV. First, the occurrence of delayed access to care and treatment was explained through pluralistic health beliefs, general health and HIV-specific knowledge and the nature of patient journeys. Second, interruptions to care and treatment was driven by availability of alternative options, failed treatment and exploitation of PLHIV by opportunistic traders and healers. Lastly, the mixing of care providers and treatment options was represented by interactions between available care providers and different forms of treatments, with bottlenecks explained by a sense of mistrust.

### Delays in accessing care and treatment

The pluralistic menu of options for healthcare which was available to PLHIV, beyond HIV clinic services, manifested in delays along the pathway of care for HIV. PLHIV, together with their family members, had decision-making powers to choose their preferred source of healthcare often sequentially, and making such decisions was considered a slow process, which in itself could cause delays. While PLHIV chose their first point of contact, be it biomedical, faith-based or traditional, the decision to maintain or change their healthcare source depended on their experience of improvement in health status. When chosen healthcare or treatment failed, PLHIV were more likely to switch sources of care, resulting in long patient journeys to HIV treatment and care.

*People do not make decisions immediately. There are many people who just start immediately with consulting a traditional (healer). After being stuck, they then decide to go and test their health. But many start with the traditional (healers), including me…* (Male, PLHIV, on ART, Tanzania)

The choice of healthcare sources depended largely on general health beliefs, whether PLHIV were more inclined towards biomedical, faith-based or traditional sources, as well as more specifically what they considered to be the type or cause of their illness. These health inclinations and explanations influenced patients’ preferred source of healthcare, which could have implications for delays in reaching the appropriate form of care and treatment.

*That is normal because we have three wings*…*we have those who believe that they Christians, we can be prayed for. When we are sick we only pray. Then we have another category which believes that if they are sick then someone is wishing them bad and they visit a traditional healer. Then the bigger percentage has realised that when they are sick, they must visit a (health) facility.* (Male, PLHIV, on ART, Kenya)

Specific knowledge and understanding of illnesses, services and treatments also influenced the extent to which PLHIV engaged in medical pluralism. Knowledge of traditional healing for PLHIV was rooted in the notion of ‘tradition’, and could be perceived as reliable when patients experienced health relief. However, awareness and knowledge, or lack thereof, of biomedical options, including HIV-specific services, facilitated access to testing and treatment. Depending on how health knowledge was relied and acted on, PLHIV could delay access to biomedical care by first consulting faith-based or traditional healers.

*There is a woman who knows traditional Haya medicines*…* I started taking those medicines*…* Um*…* it is our tradition*…* even a small child would take the medicines*…* now when I felt like my health had improved, then I told myself like: ‘You should go for testing so you may know what is troubling you.* (Male, PLHIV, on ART, Tanzania)

### Interruptions of care and treatment

Medical pluralism also contributed to bottlenecks in the HIV care cascade through interruption of biomedical care and treatment, largely manifesting as attrition from care. The use of non-biomedical medicines to cure HIV, or as an alternative to ART, was seldom reported in this study. Nonetheless, the hope for a cure among PLHIV rendered them vulnerable to false and confusing messages that came from healers who claimed to cure HIV, and instructed PLHIV to stop their HIV treatment.

*They were given holy water and instructed that if they take it for five days, they should go for re-testing, and they will see that they will be negative.* (Female, PLHIV, on ART, Zimbabwe)

The most predominant reason for people to use non-biomedical medicines after they had initiated care and treatment was the persistence of HIV or its symptoms, as declared by PLHIV, and often expressed as failure of biomedical treatment. Some patients who expected a cure would not be satisfied with the benefits of current biomedical HIV treatment options. The persistence or worsening of the illness led some patients to declare failed treatment and adopt alternative explanations such as enchantment, resulting in decisions to seek care elsewhere.

*I have taken the medicine for HIV, and the HIV is unrelenting. That only implies that this is witchcraft. That is why I kept going to the traditional doctors.* (Female, PLHIV, on ART, Uganda)

There was recognition that some health providers, particularly traders who privately sold biomedical or traditional medicines, were exploiting the vulnerability of some PLHIV for financial gain. There was further recognition that these deceitful practices hindered access to appropriate treatment, which could result in poor health outcomes following delays and interruptions.

*They visit the private clinics to buy some medicine. They also visit the traditional herbalists for help. These herbalists are not honest and in fact they take money from people. Many patients die when they fail to get the right treatment for their disease.* (Male, PLHIV, on ART, Uganda)

### Mixing of care and treatment options

Medical pluralism also contributed to bottlenecks through the practice of combining sources of healthcare and treatment options, often employed for complementary purposes. Traditional or faith-based healing was used to complement biomedical care, as seen among PLHIV who believed they were bewitched or cursed, which could lead to interactions of medicines, and providers, although often unknown to them, treating the same PLHIV.

*There are some people who take the medicine for AIDS, but they still continue to take the traditional medicine, (as) extra treatment*…* They go and start using traditional medicine alongside the hospital medicine.* (Male, PLHIV, pre-ART, Tanzania)

Some biomedical providers of HIV services feared the reality of PLHIV combining ART with other forms of non-biomedical care, due to the possibility of unwanted and unknown drug-to-drug interactions, to the extent that they denied some patients ART. These fears were also a manifestation of mistrust between biomedical providers and their non-biomedical counterparts, although the individual patients were often the ones who interacted with the different sets of providers.

*…we went to the clinic, and they said I have to start treatment, but on one condition. I have to stop traditional medicine, and use (antiretroviral) treatment. If I can’t do that, then I mustn’t start. So I have to choose one, "start the treatment if you sure you will continue because if you start and stop you will give yourself a problem". Then I chose to start.* (Female, PLHIV, on ART, South Africa)

The mixing of care options was also presented in a positive light, revealing opportunities for collaborative engagements between providers through referrals. Generally, traditional and faith-based providers referred patients to biomedical services. PLHIV reported an emerging trend of ‘progressive’ healers who were knowledgeable about HIV, and referred their patients for HIV tests before treating them. If tests were positive, they encouraged PLHIV to stay on HIV treatment prescribed by their biomedical providers. There was also recognition of a subset of biomedical providers who referred their patients to healers, although informally, following ‘failed’ biomedical treatment. These biomedical practitioners were perceived to understand traditional or faith-based aspects of illness, especially when patients recovered their health after such referrals.

*The (medical) doctor said: "It is better you take her from here and go seek some traditional medicines". I took the woman, my wife, and we went to see a traditional healer.* (Male, PLHIV, pre-ART, Tanzania)

## Discussion

This study sought to explicate the contribution of medical pluralism to bottlenecks along the HIV care cascade in eastern and southern African settings. Medical pluralism was found to influence the cascade-of-care for HIV through (1) delayed access, (2) interruptions and (3) mixing multiple sources with biomedical HIV care and treatment. Delayed access to HIV diagnosis and/or ART, often with cases of death occurring prior to treatment, has already been documented.^4 9 18^ We note here that such delays can be better understood through recognition of autonomy, beliefs and traditions held by PLHIV, and their individual, as well as collective, knowledge and experiences of chosen healthcare options available to them. The sociocultural context of medical pluralism is representative of the nature of ‘health-worlds’ within which PLHIV seek healthcare, be it traditional, faith-based or biomedical, which in turn further governs their interpretation of illness and symptoms. The concept of health-world was coined by Germond and Cochrane, drawing from Habermas’ theory of lifeworld.^19^ They argue that health-worlds relate to the empirical complexity of people’s conceptions of health, their health-seeking behaviour and their health status. Health-worlds adopt expanded definitions of medical pluralism that include faith-based options, occur at individual and social levels and are characterised by bodily experience of illness, need to solve health problems and the power relations involved.^19^

The virtual boundaries of health-worlds observed in this study provides further illumination of the ways in which PLHIV are able to switch between such health-worlds, resulting in treatment interruptions. Treatment interruptions in biomedical HIV care were often associated with failed treatment in one health-world, with the hope to find a solution through migration into another health-world. As a result, the lack of integration between health-worlds created conditions for false messages and promises of HIV cure by healers and traders who sought to exploit vulnerable PLHIV. Such conflicted and opportunistic claims have long been perceived as a source of psychological torment for both patients and their biomedical health providers,^4 20^ and blamed on the existence of unqualified traditional healers, and the lack of adequate understanding of HIV/AIDS among healers.^21–23^ Therefore, medical pluralism, irrespective of false or authentic basis, has implications for the retention of patients, and if left unaddressed, will continue to form a major bottleneck along the ‘biomedical’ cascade-of-care for HIV. Implementers of HIV services need to have a better understanding of medical pluralism and its implications for HIV care, and develop more effective ways of engaging with people’s health behaviours and preferences, so as to increase chances of HIV care and treatment success.

PLHIV were also able to straddle two health-worlds simultaneously, through concurrent use of biomedical and traditional/spiritual healthcare in a complementary rather than alternative manner, and therefore not intended to replace ART.^24^ In such cases, medical pluralism is adopted to supplement the care received, in order to fulfil additional healthcare needs, such as other psycho-socio-spiritual needs catered for by the combination of healthcare systems.^4^ The health-worlds of PLHIV who engage in concurrent medical pluralism can be better understood by making a distinction between the notion of enchantment as a distal cause of their illness, and the virus as the biological proximal cause of the disease, both of which require intervention.^23 25 26^ While individual or shared beliefs, either traditional or faith-based, serve to explain people’s engagement in medical pluralism, many such beliefs are seen by others, including biomedical practitioners, as ignorant.^20 27^ Such explanations are not well-received among biomedical practitioners, who are said to know little about medical pluralism, often resulting in a conflict of healthcare systems.^28 29^ These notions of tension were challenged in our study by the practices of some biomedical practitioners who recognised the need for and ‘referred’, although informally, PLHIV to the traditional healthcare system. In so doing, they were seen to endorse or approve of medical pluralism by their patients. Nevertheless, the reluctance of biomedicine to embrace medical pluralism, may discourage plural healthcare users from disclosing such practices to their HCWs, and potentially compromise the effectiveness of biomedical interventions.^28 30^

In conclusion, this multicountry study demonstrates that medical pluralism, as a common form of healthcare behaviour among PLHIV, acts through mechanisms of delays, interruptions and mixing care and treatment, causing bottlenecks to healthcare along the HIV cascade in sub-Saharan Africa. This problem necessitates a collective agenda to design interventions intended to address various mechanisms through which bottlenecks occur as a result of medical pluralism. However, the biggest challenge facing medical pluralism intervention efforts relate to the reluctance of biomedical healthcare systems to embrace the notion of medical pluralism. Users of dual systems of healthcare appear to resolve such tensions for themselves by justifying the need for and benefit of their respective healthcare systems of choice. In which case, the combination of the two systems suggests that traditional healthcare may compensate for PLHIV perceived shortfalls and treatment failures in the biomedical healthcare system, or vice versa. There is therefore a need for biomedical systems and providers to recognise and acknowledge the role and importance of context, including sociocultural and spiritual beliefs, practices and traditions, and the management of PLHIV within the health-worlds. Such recognition may create possibilities for different medical systems to work collaboratively to foster a cohesive form of medical pluralism, which functions in the best interest of plural users living with HIV.

**Key messages**

- In sub-Saharan Africa, medical pluralism involves ‘shopping and switching’ between multiple modalities of care available, including biomedical, traditional and faith-based healthcare systems.
- Medical pluralism contributes to bottlenecks along the HIV care cascade through delays and interruptions of care, as well as tensions and mistrust between healthcare providers.
- Culture-sensitive interventions and policies designed to minimise the potential harm and consequences of medical pluralism for PLHIV are urgently needed in sub-Saharan Africa.
